# Draft Genome Sequence of a Clostridioides difficile Sequence Type 97 Strain Belonging to Hypervirulent Clade 2

**DOI:** 10.1128/MRA.00245-20

**Published:** 2020-04-02

**Authors:** Yukino Usui, Yoko Nukui, Ryuji Koike, Shuji Tohda, Ryoichi Saito

**Affiliations:** a Department of Molecular Microbiology, Graduate School of Medical and Dental Sciences, Tokyo Medical and Dental University, Tokyo, Japan; b Department of Infection Control and Prevention, Tokyo Medical and Dental University Medical Hospital, Tokyo, Japan; c Department of Clinical Laboratory, Tokyo Medical and Dental University Medical Hospital, Tokyo, Japan; University of Rochester School of Medicine and Dentistry

## Abstract

Clostridioides difficile hypervirulent clade 2 lineages are the major lineages responsible for the outbreaks in North America and Europe. However, the genome sequences of Japanese isolates are scarcely available. Herein, we report the draft genome sequence of C. difficile strain TMD0138 sequence type 97 (ST97), belonging to hypervirulent clade 2, isolated in Japan.

## ANNOUNCEMENT

The morbidity and severity of Clostridioides difficile infections have increased since the 2000s in both health care facilities and communities (1). The epidemic hypervirulent strain known as ribotype 027 (RT027)/sequence type 1 (ST1), belonging to clade 2, is characterized by the production of binary toxins, increased production of toxins A and B owing to an 18-bp deletion in *tcdC*, hypersporulation, and high-level fluoroquinolone resistance (1–3). However, in comparison to North America and Europe, whole-genome sequence data for Japanese clade 2 lineages are extremely scarce, including the data published in our previous report (4). In this study, we report the draft genome of the community-acquired strain C. difficile TMD0138, belonging to hypervirulent clade 2, RT027/ST97, recovered from a 31-year-old male patient admitted to the Medical Hospital of Tokyo Medical and Dental University in 2001 (5).

C. difficile strain TMD0138 was grown in brain heart infusion (BHI) broth supplemented with 5 g/liter yeast extract and 0.1% (wt/vol) l-cysteine and incubated at 37°C under anaerobic conditions for 7 h. Genomic DNA was extracted using a NucleoSpin tissue kit (TaKaRa Bio) and then quantified with a QuantiFluor double-stranded DNA (dsDNA) system (Promega). A library was prepared from 50 ng of fragmented DNA using a KAPA HyperPrep kit (Kapa Biosystems) and then sequenced with the NextSeq platform (Illumina) to produce 2 × 151-bp paired-end reads. A total of 3,730,693 reads (average length, 150 nucleotides) were generated, following which quality control was performed on the raw reads using Sickle v. 1.33 (https://github.com/najoshi/sickle). The reads were assembled *de novo*, using SPAdes v. 3.10.1 (6), into 104 contigs, larger than 200 bp (largest scaffold, 483,781 bp; *N*_50_, 196,318 bp; *L*_50_, 8; average GC content, 28.44%), with an estimated genome size of 4,132,232 bp and an average coverage of >100×. Default parameters were used for all software.

*In silico* multilocus sequence typing (MLST) performed by MLST v. 2.0 (7) assigned strain TMD0138 to ST97, which belongs to clade 2. A previous study demonstrated that TMD0138 belongs to the same strain characterized as RT027 by PCR ribotyping (5). Comparative analyses of the pathogenicity locus (PaLoc) and Cdt locus (CdtLoc) sequences in TMD0138 were conducted by BLAST and ClustalW v. 2.1. The PaLoc sequence was homologous to reference genome 630, belonging to RT012/ST54 (GenBank accession number NC_009089; 100% query coverage and 96.57% identity), and strain R20291, belonging to RT027/ST1 (GenBank accession number FN545816; 100% query coverage and 99.83% identity). The negative regulator gene, *tcdC*, in TMD0138 was 99.41% identical to that in R20291, with 3 nucleotide substitutions, but lacked the single-nucleotide deletion at position 117. The CdtLoc sequence in TMD0138 showed 99.88% identity with that of R20291, and the binary toxin regulator gene, *cdtR*, in TMD0138 was completely conserved with that of R20291.

PHASTER v. 4.3X (8) identified two intact prophage regions (scores, >90), the C. difficile bacteriophages phiMMP03 and phiMMP01. The genome of TMD0138 also possessed an incomplete prophage region, and the most common phage was phiCDHM19 (score, 40).

### Data availability.

This whole-genome shotgun project has been deposited at DDBJ/ENA/GenBank under BioProject number PRJNA530817 and accession number WUUI00000000. The raw data are available under SRA accession number SRR11241614.

## References

[1] Fatima R, Aziz M 2019 The hypervirulent strain of *Clostridium difficile*: NAP1/B1/027—a brief overview. Cureus 11:e3977. doi:10.7759/cureus.3977. 30967977 PMC6440555

[2] Warny M, Pepin J, Fang A, Killgore G, Thompson A, Brazier J, Frost E, McDonald LC 2005 Toxin production by an emerging strain of *Clostridium difficile* associated with outbreaks of severe disease in North America and Europe. Lancet 366:1079–1084. doi:10.1016/S0140-6736(05)67420-X. 16182895

[3] Akerlund T, Persson I, Unemo M, Noren T, Svenungsson B, Wullt M, Burman LG 2008 Increased sporulation rate of epidemic *Clostridium difficile* type 027/NAP1. J Clin Microbiol 46:1530–1533. doi:10.1128/JCM.01964-07. 18287318 PMC2292905

[4] Saito R, Usui Y, Ayibieke A, Nakajima J, Prah I, Sonobe K, Aiso Y, Ito S, Itsui Y, Hadano Y, Nukui Y, Koike R, Tohda S 2019 Hypervirulent clade 2, ribotype 019/sequence type 67 *Clostridioides difficile* strain from Japan. Gut Pathog 11:54. doi:10.1186/s13099-019-0336-3. 31700548 PMC6827173

[5] Sawabe E, Kato H, Osawa K, Chida T, Tojo N, Arakawa Y, Okamura N 2007 Molecular analysis of *Clostridium difficile* at a university teaching hospital in Japan: a shift in the predominant type over a five-year period. Eur J Clin Microbiol Infect Dis 26:695–703. doi:10.1007/s10096-007-0355-8. 17647032

[6] Bankevich A, Nurk S, Antipov D, Gurevich AA, Dvorkin M, Kulikov AS, Lesin VM, Nikolenko SI, Pham S, Prjibelski AD, Pyshkin AV, Sirotkin AV, Vyahhi N, Tesler G, Alekseyev MA, Pevzner PA 2012 SPAdes: a new genome assembly algorithm and its applications to single-cell sequencing. J Comput Biol 19:455–477. doi:10.1089/cmb.2012.0021. 22506599 PMC3342519

[7] Larsen MV, Cosentino S, Rasmussen S, Friis C, Hasman H, Marvig RL, Jelsbak L, Sicheritz-Ponten T, Ussery DW, Aarestrup FM, Lund O 2012 Multilocus sequence typing of total-genome-sequenced bacteria. J Clin Microbiol 50:1355–1361. doi:10.1128/JCM.06094-11. 22238442 PMC3318499

[8] Arndt D, Grant JR, Marcu A, Sajed T, Pon A, Liang Y, Wishart DS 2016 PHASTER: a better, faster version of the PHAST phage search tool. Nucleic Acids Res 44:W16–W21. doi:10.1093/nar/gkw387. 27141966 PMC4987931

